# The Age of Mobility: Can Equalization of Public Health Services Alleviate the Poverty of Migrant Workers?

**DOI:** 10.3390/ijerph192013342

**Published:** 2022-10-16

**Authors:** Ziming Zhou, Yumeng Jiang, Haitao Wu, Fan Jiang, Zhiming Yu

**Affiliations:** 1 School of Business Administration, Zhongnan University of Economics and Law, Wuhan 430073, China; 2 Institute of Dermatology, Chinese Academy of Medical Sciences and Peking Union Medical College, Nanjing 210042, China; 3 Institute of Agricultural Economics and Technology, Hubei Academy of Agricultural Science, Wuhan 430073, China

**Keywords:** migrant workers, public health services, multidimensional poverty, heterogeneity analysis, PSM

## Abstract

Migrants workers are important participants in and contributors to economic and social construction, but they still face the reality of being marginalized. Based on data from the China Migrants Dynamic Survey in 2018, this paper systematically investigated the impact of public health services on the multidimensional poverty of migrant workers. The research found that, first, the current mean of the multidimensional poverty deprivation value of migrant workers is 0.1806, which is one dimension of poverty that exists on average. In addition, migrant workers do not have high access to public health services. The proportions of migrant workers who have not established residents’ health files and who have not received public health education are 74.22% and 29.92%, respectively. Second, public health services can significantly alleviate the multidimensional poverty of migrant workers. After mitigating the potential endogeneity problem by the IV-2SLS method and conducting robustness tests by the PSM method, the conclusion is still robust. Further research found that the impact of public health services on the multidimensional poverty alleviation of migrant workers is heterogeneous. The improvement of public health services has the greatest effect on the multidimensional poverty alleviation of the new generation of migrant female workers in the western region. The research in this paper helps to examine and clarify the policy significance of public health services for the multidimensional poverty alleviation of migrant workers and provides empirical evidence for the use of public health services to tackle the poverty problem.

## 1. Introduction

Migrant workers are important participants and contributors to economic and social construction. Since the reform and opening-up, hundreds of millions of farmers have moved from rural areas to cities, becoming the backbone of the labor market in urban areas and making great contributions to sustained economic growth. The migrant worker monitoring survey report released by the National Bureau of Statistics shows that the total number of migrant workers nationwide in 2020 reached 285.6 million, of which 169.59 million migrant workers left their local area, accounting for more than 59% (2020 Migrant Worker Monitoring Survey Report. http://www.stats.gov.cn/tjsj/zxfb/202104/t20210430_1816933.html, accessed on 1 August 2022).

Judging from the development experience of various countries in the world, with the continuous advancement of urbanization, the level of social welfare enjoyed by residents has gradually increased. The improvement of people’s welfare in the urbanization process is in line with the general development law [1]. However, compared with urban residents, migrant workers are characterized by high mobility, coupled with low income levels and lack of medical insurance, often underestimating health risks and with poor awareness of disease prevention and control. The work characteristics and social roles of migrant workers determine the vulnerability of their urban survival and development, and they are more susceptible to the impact and disturbance of diseases and loneliness [2]. Such noninstitutional factors, including the inertia of the traditional rules of resource allocation and the quality of migrant workers, restrict the ability of migrant workers to enter cities and their development space, making them more likely to fall into the predicament of urban integration, being in the middle of urban and rural survival, and becoming a major group of the new urban poor [3].

Poverty reduction is affected by many factors, such as policies, the environment, individuals, and families. An increase in the level of public service supply will help reduce the inequality brought about by the operation of market mechanisms and will also help narrow the economic gaps among regions, industries, and social groups [4]. Basic public health services are a major institutional arrangement for deepening the reform of China’s health care system and promoting the “Healthy China” strategy. Because of its obvious “public attributes”, it has a specific internal connection with poverty reduction [5]. In addition, public health services also have a greater “enabling” effect than other public services in reducing poverty [6].

Since the deepening of the medical system reform in 2009, a number of policies have been issued for the construction of a high-quality and efficient public health service system, especially emphasizing the importance of public health services to the migrant population and helping migrant workers avoid health risks and economic risks [7]. However, there are also opinions that migrant workers who enter cities are restricted by factors such as the household registration system and its derived welfare system and land system, as well as the human capital and social capital of the floating population. The territorial characteristics and urban bias generated by basic public services exclude migrant workers from the urban basic public services and social welfare security system [8,9], increase the health costs of migrant workers in the city, and, to a certain extent, seriously damage their welfare [10,11].

In this context, based on the China Migrants Dynamic Survey (CMDS) data organized by the National Health Commission, this paper systematically evaluates the impact of public health services on migrant workers from the two aspects of public health education and health file registration. The impact of multidimensional poverty and the heterogeneity of this impact are analyzed. The innovation of this paper is to focus on the relationship between service accessibility and poverty among migrant worker groups from the perspective of public health services. It provides new ideas to improve migrants’ sense of well-being and promote integration into the city. It also provides some references for other countries in the world to explore the reform of poverty alleviation mechanisms and policy innovations to improve the efficiency of public health services. To achieve this vision, the message needs to be communicated not only to migrant workers, but also to the broader policy community. We must take action to improve the health and poverty of migrant workers. Therefore, we call for improved public health policies to adapt to the global and national health challenges of this new era.

We organize the rest of the paper as follows: Section 2 describes the theoretical and empirical framework; Section 3 describes our data and choice of regression procedures; Section 4 and Section 5 report and discuss our empirical results; and Section 6 concludes.

## 2. Theoretical and Empirical Framework

**Figure 1:**
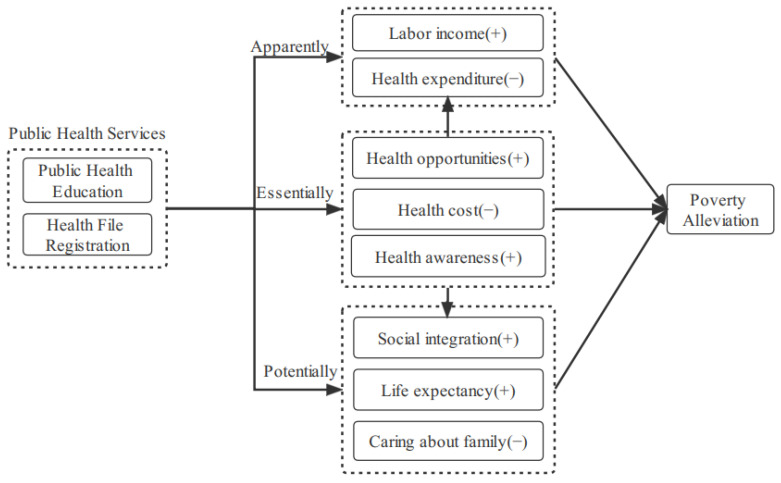
The theoretical logic of the influence of public health services on the poverty of migrant workers.

Public service is the basic function of modern government. Compared with the state’s high attention to the top-level design of public services and the rapid advancement in practice, standardized and in-depth academic research in the field of public health services is slightly lagging. For a long time, the “siphon effect” in the allocation of health resources in China has caused the efficiency of health resources to be in a state of continuous overflow or diminishing returns from governance [12]. Whether from the perspective of overcoming market deficiencies or reducing economic inequality, public health services can also help correct the results of the operation of market mechanisms [13]. Existing research on the positive externalities brought about by public health services is mainly carried out from the following three aspects (see Figure 1).

### 2.1. The Essential Impact of Public Health Services on Health Poverty

Health is the human capital for individuals to obtain social resources and achieve self-development and is an important guarantee for the healthy operation of the family and the entire society. The health-improving effect of public health services can be mainly launched from the following three aspects. One is to protect health opportunities. Inequality in the allocation of health resources directly restricts the development of residents’ health capabilities. Public health services can improve the efficiency of medical resource allocation by improving the accessibility of medical services, thereby becoming an important opportunity and condition for migrant workers to reduce their health and poverty [14]. Second, public health services reduce health costs. The migrant worker group is a mobile group, and social fragility limited by the ability to obtain social resources prevents individuals from paying for the cost of health interventions and thus allows them to fall into a state of health poverty due to the lack of health interventions. Public health services not only help to reduce the direct cost of residents’ access to health resources but also reduce the indirect costs of transportation, accommodation, lost work, and even unemployment caused by residents’ restricted access to health resources [15]. Third, public health services raise health awareness. The increase in health awareness helps to reduce the health risk factors for migrant workers. Because public health services are free and convenient, they help migrant workers transform from a passive health resource input to an active health opportunity acquisition. This helps migrant workers actively refuse to work overtime, which is harmful to their health and reduces the phenomenon of exchanging health for income [16].

### 2.2. The Apparent Impact of Public Health Services on Economic Poverty

Regarding basic public health service items, the existing literature has mostly carried out research on some regions or populations, and its positive health improvement effect has reached a consensus in the academic community [17]. On this basis, public health services will have an “open source” effect on the economic level of migrant workers. This is because there is a positive circulation mechanism between the health and income of migrant workers [18]. Healthy capital in human capital can not only increase labor participation and nonagricultural employment opportunities but also increase people’s labor productivity in the long run [19]. Therefore, public health services can increase the health capital of migrant workers, thereby increasing their labor income [20]. At the same time, the improvement of health levesl will also reduce the precautionary savings of migrant workers in response to uncertainty, which will help to further increase their productive investment and human capital investment. On the other hand, public health services have a “throttling” effect. The health risks of migrant workers can be reduced through free medical education and health promotion, which also means that their economic risks are reduced, income and expenditure fluctuations caused by health risks are reduced, and the economic poverty of migrant workers can be indirectly improved.

### 2.3. The Potential Impact of Public Health Services on Psychological Poverty

The classic theory of sociology reminds us that in the process of urban migration, migrant workers will not only encounter the problem of adapting to urban life but also encounter the problem of exclusion from urban public services [21]. Wages, housing, children’s education, medical care, and returning home are the most important concerns of migrant workers. The study found that the proportion of negative emotions, such as worrying, complaining, and impatience, among migrant workers has increased significantly [22]. Under the influence of the mentality of being a marginalized people, inferiority and closedness are common among migrant workers [23]. Empirically, migrant workers’ mobility is roughly divided into three stages, in the order of individual or couple migration, nuclear family migration, and main family migration. This also means that at the beginning of the migration, migrant workers had to move between urban and rural areas in the form of family separation, not only to solve the problem of social integration but also to consider their future development and the problems of left-behind elderly, women, and children in their hometowns [24]. This also causes migrant workers to not only lack a sense of security in social integration but also to lack a sense of security in life expectations and a lack of satisfaction in family concerns [25,26]. The increase in the openness of urban public health services, on the one hand, affects the transformation of migrant workers’ values, lifestyles, and behaviors and directly reduces the cost of urban living on the health of migrant workers [27]. On the other hand, it helps to shorten the psychological distance from the city and enhance the identity and social integration of migrant workers [28].

## 3. Materials and Methods

### 3.1. Research Sample

#### 3.1.1. Data

The data used in this paper is the “2018 China Floating Population Dynamics Monitoring Survey Data” (CMDS2018). There are three main reasons for choosing this set of data. First, there is the combination of authority and reliability. The CMDS2018 data are organized and developed by the National Health Commission and implemented by the China Population and Development Research Center and the China Health Education Center. The sample covers 31 provincial-level administrative units and the Xinjiang Production and Construction Corps and strictly implements a stratified, multistage, and scale-proportional PPS sampling method. The primary sampling unit is the township (town, street). The scope of the survey covers 7600 sample points. There are nearly 170,000 annual samples. Second, there is the combination of professionalism and applicability. The object of the CMDS2018 data survey is the inflow population who has lived in the inflow area for one month or more and is not registered in the district, aged 15 and above. The content covers the income and expenditure of the migrant population, employment, mobility and willingness to stay, health, social integration, and other aspects. (The total target population did not include the transient inflow of people at stations, docks, airports, hotels, hospitals, etc. at the time of the survey, and it did not include the mobile population with the status of “school students”. For the inflow of people who meet the overall sampling requirements but live in informal places (temporary workplaces, abandoned factories, roadsides, etc.), a sub-sampling frame is prepared). Third, there is the high time-sensitivity of the data. The CMDS2018 data are the latest survey data released by the National Health Commission. This source has strong timeliness and completeness and can better meet the data requirements of this article. Based on the sample research needs of this paper and the consensus of experts, this paper only considers the sample of the migrant population with agricultural household registration who migrated to work or do business and were employed in CMDS2018. After processing the missing values and outliers of each variable, a benchmark sample containing 53,857 observations was obtained.

#### 3.1.2. Variables

Poverty deprivation levels. The formation mechanisms and manifestations of poverty are diverse, connected, and heterogeneous. Any attempt to define and operationalize poverty has certain limitations [29]. Although there is no uniform standard or measurement scale for the dimensions and indicators of multidimensional poverty, the existing research literature shows that the more widely used dimensions include income, health, education, medical care, and quality of life. This article considers both the availability of data and the characteristics of the migrant workers themselves while taking into account that most of the migrant workers have a fixed level of education and are highly nonreversible. Therefore, this article does not include education in the research dimension of migrant workers’ multidimensional poverty but innovatively increases the two dimensions of migrant workers’ life expectancy and their family concerns. This paper has designed a multidimensional poverty evaluation index system for migrant workers that includes 5 dimensions and 11 indicators (See Table 1). From the perspective of the deprivation of rights, referring to existing research, the deprivation of rights in any dimension should be taken seriously. Therefore, this article adopts an equal weight method to empower each dimension.

Public health service. The Pilot Program for Equalization of Basic Public Services for Health and Family Planning for the Floating Population, issued by the National Health Commission in 2013, provides more than 12 public health services. Carrying out public health education and implementing health file registration for the floating population are the key tasks in the content of public health services, and they serve the largest number of groups. Therefore, this article takes “public health education” and “health file registration” as the core explanatory variables. Among these, “public health education” asks about the number of migrant workers who have received nine aspects of health education in the past year, namely, occupational disease prevention and control, STD/AIDS prevention and control, reproductive health and contraception, tuberculosis prevention and control, smoking control, mental health, chronic disease prevention and treatment, maternal and child health care/prenatal and postnatal care, and self-help in public emergencies. “Health file registration” asks migrant workers whether they have an established resident health file in their current residence.

Control variables. To improve the effectiveness of the estimation and to try to avoid the estimation error caused by the omitted variables, combined with the existing research and related theoretical basis, this paper mainly determines the three types of control variables from the characteristics of human capital, family characteristics, and regional characteristics. At the same time, the article controls the industry fixed effects (state-owned enterprises, government institutions, private enterprises, individual industrial and commercial households, other departments, and freelancer) and the fixed effects of outflow areas (rural, township, county, prefecture-level city, municipality directly under the central government, and provincial capital city).

### 3.2. Methods

#### 3.2.1. Benchmark Model

The main relationship we want to explore in this study is whether the equalization of public health services would alleviate the poverty of migrant workers. To this end, we estimate Equation (1):

(1) $$Poverty_{i}=\beta_{0}+\beta_{1}PHS_{i}+\delta X_{i}+\varepsilon_{i}$$

On the right-hand side of Equation (1), $PHS_{i}$ is public health service items, $X_{i}$ represents the control variable that affects the multidimensional poverty level of migrant workers, $\beta_{0}$, $\beta_{1},$ and δ are the parameters to be estimated, and $\varepsilon_{i}$ is the random disturbance term.

One may be concerned that this paper uses non-experimental data. Considering whether migrant workers enjoy urban public health services (whether they have established health files and received public health education) may not satisfy random sampling, and direct regression may lead to a sample self-selection problem. Therefore, this paper will use the propensity score matching method (PSM) to construct a counterfactual framework to alleviate the sample self-selection problem. The basic idea of the PSM method lies in dividing the whole sample into treatment groups and reference groups based on whether they have access to urban public health services, predicting the probability of migrant workers enjoying public health services based on observable variables, and using methods such as nuclear matching to identify control group variables. This forms a matching sample to achieve results similar to the effect of random trials to solve the possible impact of the above problems [30].

Using a logit model to estimate the probability of migrant workers accessing public health services (public health education or health file registration) based on existing literature and theory, we can obtain the propensity value:

(2) $$P\left(X_{i}\right)=Pr\left(F_{i}=1|X_{i}\right)=\frac{exp\left(\beta X_{i}\right)}{1+exp\left(\beta X_{i}\right)}+\varepsilon$$

The dual dummy variable F in the formula represents migrant workers’ enjoyment of public health services, $X_{i}$ represents the influencing factors of migrant workers’ enjoyment of public health services, β is the coefficient of the model, and ε is the random disturbance term.

(3) $$ATT=E\left(Y_{i,1}|T_{i}=1\right)-E\left(Y_{i,0}|T_{i}=1\right)$$

#### 3.2.2. Discussion of Endogenous Problems

The prerequisite for the effectiveness of the PSM model is to satisfy the assumption of negligibility; that is, all individual characteristics or related variables used for matching are included in the regression equation, and there are no missing variables related to explanatory variables (whether migrants have access to public health services). However, this is a strong assumption. Although this article has controlled as many covariates as possible, there may still be endogeneity problems caused by missing variable deviations or measurement errors. At this time, the potential results (poverty alleviation effects) will not be independent. Whether migrants have access to public health services is likely to bias the estimation results. To this end, this article uses the IV-2SLS method to further verify the robustness of the results.

The IV-2SLS method requires two stages of regression; that is, before estimating Formula (1), the first stage is needed to find the exogenous part of the endogenous variable. Specifically, in this article, the first-stage regression equation is as follows:

(4) $$PHS_{i}=\partial_{0}+\partial_{1}IV_{i}+\delta X_{i}+\varepsilon_{i}$$

Among these, $PHS_{i}$ is the core explanatory variable, which represents the level of migrant workers enjoying public health services. $IV_{i}$ is an instrumental variable; $X_{i}$ is a control variable; $\partial_{0}$, $\partial_{1}$, and δ are estimated parameters; and $\varepsilon_{i}$ is a random disturbance term. The IV-2SLS model regards core explanatory variables as endogenous variables, and instrumental variables need to be added to the first stage of regression. An effective instrumental variable needs to meet the correlation condition, that is, be related to the core explanatory variables. At the same time, it also needs to meet the exogeneity condition, that is, it is not directly related to the explained variable.

This article chooses two instrumental variables. First, from the perspective of the regional policy environment, according to the two questions in the questionnaire, “the number of health/fertility/health education bulletin boards in the village where the migrants are located/the center is fixed” and the “number of bulletin board updates in the past year”, we take the logarithm of the product of the two to construct a new variable “community public health attention” as an instrumental variable for whether migrant workers receive public health services. On the one hand, “community public health attention” reflects to a certain extent the publicity level and service intensity of public health services for migrant workers in the community and can directly affect the choice of migrant workers to receive public health services. On the other hand, “community public health attention” will not have a direct impact on the multidimensional poverty level of migrant workers.

Second, from the perspective of migrant workers’ policy cognition, the questionnaire question “Have you heard of public health service items?” was selected as an instrumental variable. The “policy cognition” of migrant workers on public health services will affect whether they accept public health services, but this level of cognition does not have a direct impact on their own multidimensional poverty levels. Logically speaking, the “community public health attention” and “policy cognition” of migrant workers meet the conditions of relevance and exogeneity and are effective instrumental variables.

Specifically, in this paper, to test the validity of the IV-2SLS estimation results, we carried out under-recognition tests, weak recognition tests, and overidentification tests on the instrumental variables. This article refers to the research of Staiger and Stock (1997). The F value of the first stage of the IV-2SLS model is above 10, indicating that there is a strong correlation between instrumental variables and endogenous variables [31]. Regarding whether the instrumental variables meet the exogenous condition, this paper adopts the overidentification test of instrumental variables, which is consistent with expectations. The *p* values of the overidentification test are all greater than 0.1, indicating that we cannot reject the null hypothesis that the instrumental variables meet the exogenous condition. It can be seen that the instrumental variables in this article also meet the exogenous conditions.

## 4. Estimation Results

### 4.1. Statistical Analysis

Table 2 reports the multidimensional poverty level of migrant workers. The average value of the multidimensional poverty deprivation value of migrant workers is 0.1806, and 34.75% of migrant workers are in a state of zero deprivation, nearly 65% of migrant workers are still in varying degrees of deprivation. Specifically, from the perspective of each dimension, the average poverty incidence of the life expectancy dimension and the economic dimension is the highest, 27.11% and 26.85%, respectively. The average incidence of poverty in the health dimension and the dimension of caring about family is 15.64% and 13.81%, respectively. The average incidence of poverty in the social integration dimension is the lowest, only 6.87%.

The level of public health service for migrant workers: Overall, only 25.78% of migrant workers established residents’ health files in their current residences, and 29.92% of migrant workers did not receive any kind of public health education. Specifically, among the nine public health education items involved in the CMDS questionnaire, the highest proportion of migrant workers received health education on smoking control, accounting for approximately 49.96%. Second, they received health education on reproductive health and contraception and maternal and child health/prenatal and postnatal care, accounting for 46.17% and 45.67%, respectively. A total of 42.01% of migrant workers received health education on self-help in public emergencies. In contrast, there are five types of public health education that received less than 40%. The proportion of migrant workers who received health education on tuberculosis prevention and treatment is the lowest, only 30.85%. The proportion of migrant workers who received mental health education is 33.52%. The proportions of health education in the prevention and treatment of chronic diseases and occupational diseases are 34.07% and 34.15%, respectively. In addition, the proportion of people who received health education on STD/AIDS prevention is not high, accounting for only 37.18%.

### 4.2. Benchmark Regression Results

First, this paper uses OLS regression to estimate the poverty reduction effect of public health services on migrant workers. The results in Table 3 show that Columns (1) and (2) take public health education and health file registration as the core explanatory variables. When controlling the fixed effects of industries and outflow areas, it is found that public health services and the multidimensional poverty of migrant workers present a significant negative correlation. On this basis, Columns (3) and (4) further add the control variables of personal human capital characteristics, family characteristics, and regional characteristics, and the results obtained are still significantly negative. Specifically, for every additional item of public health education that migrant workers receive, their multidimensional poverty level will be reduced by 0.14%. If migrant workers receive all health education, their poverty deprivation levels will be reduced by 1.26%, approaching the poverty-reducing effects of education. Similarly, the establishment of health files will reduce the poverty deprivation levels of migrant workers by 1.62% compared to migrant workers who have not established health files. Therefore, the group of migrant workers receiving public health services developed by the community has a significant effect on alleviating their own poverty deprivation levels.

### 4.3. Endogenous Problems

Table 4 reports the regression results of the IV-2SLS method. The one-stage F statistics in Column (1) and Column (3) are 722.31 and 601.84, respectively, and both reject the null hypothesis of weak instrumental variables. At the same time, judging from the estimation results, the two instrumental variables “community public health attention” and “policy cognition” of migrant workers have a significant positive impact on public health education and health file registration. This also further demonstrates that the instrumental variables and the endogenous explanatory variables meet the correlation condition. On the other hand, the *p*-value of the overidentification test are 0.5901 and 0.7667, respectively, and the null hypothesis cannot be rejected, indicating that the instrumental variables meet the exogenous condition. According to the results of the IV-2SLS estimation in Columns (2) and (4), under the control of all control variables, fixed industry effects and fixed effects of outflow areas, public health services still have a significant negative impact on the multidimensional poverty of migrant workers. Moreover, the estimated value of the coefficient is larger than the estimated value of the OLS regression coefficient, indicating that the previous benchmark regression underestimated the poverty reduction effect of public health services on migrant workers.

### 4.4. Robustness Test

#### 4.4.1. Alleviate the Sample Self-Selection Problem

First, the propensity score of migrant workers to obtain public health services is calculated. Then, it is counterfactual to look for the work of farmers who do not have public health services who have the closest score to the migrant workers who have access to public health services. Finally, we compare the differences in the probability of falling into multidimensional poverty between the two groups and take the average of the calculated differences to obtain the average effect of public health services on the poverty reduction of migrant workers. However, the PSM method is usually suitable for cases in which the core explanatory variable is a binary variable. Although the variable of health file registration meets the requirements, the variable of public health education still needs to be dealt with. In view of this, this article attempts to adopt two methods to adjust the public health education variable into a binary variable.

First, in terms of quantity, if migrant workers have received at least one category of public health education, the value of public health education is 1, and if they have not received any public health education, the value is 0. On the other hand, considering that there are nine categories of public health education dimensions, this paper takes the median. If migrant workers have received five or more categories of public health education, the value is 1; otherwise, the value is 0. Second, CMDS2018 involves a total of nine public health education programs in terms of the classification of public health education, but there are significant differences in the importance of different education programs for migrant workers. Considering that the working environment of migrant workers is often closely related to “noise”, “dust”, and “high temperature”, there are often occupational diseases (such as pneumoconiosis, blood diseases, poisoning, etc.) suffered by migrant workers [32]. Therefore, in this paper, whether migrant workers have received health education on occupational disease prevention is used as a measure of whether migrant workers have received public health services. The value of occupational disease prevention and control is assigned as 1; otherwise, the value is 0. At the same time, we take into account the impact of recent floods and droughts caused by climate change, production accidents, and public health emergencies caused by the new coronavirus pneumonia epidemic on the production, life, and even life safety of migrant workers [33]. This article also takes into account whether migrant workers have received health education on self-rescue in public emergencies. The value of migrant workers’ health education in self-help in public emergencies is 1; otherwise, the value is 0.

Table 5 reports the average treatment effect (ATT) obtained after correcting the selection bias using the PSM method. Overall, public health services have a significant poverty reduction effect on migrant workers, a finding that is consistent with the previous results. In terms of public health education, although the ATT obtained by different methods of public health education is different, they are all negatively significant. Specifically, in terms of coefficients, receiving health education on occupational disease prevention and control has a more obvious effect on the poverty alleviation of migrant workers. Similarly, the ATT value of health file registration is −0.0212, and it is significant at the 1% significance level, indicating that health file registration has a significant poverty reduction effect on migrant workers, which further verifies the robustness of the previous conclusions.

#### 4.4.2. Use Binary Explanatory Variables

When setting the variable of multidimensional poverty in the paper, more consideration is given to the deprivation value, so multidimensional poverty is a continuous variable. Further, we use the binary variables “poverty status” and “difficult status” as explanatory variables for robustness tests in this section. For the poverty status, we set the multidimensional poverty dimension threshold to 2. A migrant worker is in poverty if he exists as deprived in more than two dimensions. For the difficult status, we refer to the problem “Is your family in the local area currently in difficulty?” in the questionnaire. If the migrant worker is experiencing hardship, he is in a difficult status. The regression results using the instrumental variable method are shown in Table 6. The results show that receiving public health education and establishing health files have a significant alleviation effect on the multidimensional poverty level of migrant workers, which further verifies the robustness of the core conclusions.

## 5. Discussion

As mentioned earlier, the abovementioned studies confirmed that access to urban public health services can significantly reduce the multidimensional poverty of migrant workers. However, the above results are only average effects. Affected by various factors, such as regional policies, economic development level, and individual characteristics of migrant workers, the impact of public health services on the poverty of migrant workers may have regional differences and intragroup differences. Therefore, it is necessary to start from gender heterogeneity, intergenerational heterogeneity, and regional heterogeneity to explore the possible heterogeneous impact of urban public health services on the poverty alleviation of migrant workers with different characteristics.

Gender Heterogeneity. Table 7 demonstrates the impact of public health services on the multidimensional poverty of migrant workers of different genders. The results show that, compared with male migrant workers, receiving public health education or setting up health file registration has a greater impact on female migrant workers’ poverty reduction. A possible explanation is that due to the social status of migrant female workers in cities and their relatively disadvantaged position in the labor market, their labor intensity often exceeds their physical health tolerance [34]. However, influenced by traditional Chinese thoughts, Chinese women often rank their health after their husbands and children and still give priority to the elderly, children, and husbands in access to medical services and health protection [35]. Although female migrants have a stronger willingness to invest in health than men, they spend less on medical care than men [36]. Therefore, the free public health intervention measures provided by the state will significantly improve the health, social integration and future development of migrant female workers, thereby reducing their multidimensional poverty levels [37].

Intergenerational Heterogeneity. There may also be differences in the impact of public health services on migrant workers across generations. Referring to the 2013 National Migrant Workers Monitoring Survey Report by the National Bureau of Statistics (http://www.stats.gov.cn/tjsj/zxfb/201405/t20140512_551585.html, accessed on 1 August 2020), migrant workers born in 1980 and later are classified as the new generation, whereas those born before 1980 are categorized as the old generation [38]. As shown in Table 8, in terms of generations, compared with the old generation of migrant workers, public health services have a greater impact on the poverty reduction of the new generation of migrant workers, but this gap is limited. This may be because the capital reserves of the new generation of migrant workers are relatively insufficient, and the network resources are not as good as those of the old generation. Therefore, considering factors such as health costs, the new generation of migrant workers needs more attention and assistance from cities, and the demand for public health services will be greater. On the other hand, the migrant workers of the new generation generally have a higher education level, and due to age factors, their health level is generally better than that of the old generation [39]. This has reduced the effect of public health services on its multi-dimensional poverty alleviation to a certain extent.

Regional Heterogeneity. Public health services are government-led. Considering that different regions in China differ in terms of economic development level, government governance capacity, and resource endowment of migrant workers, the baseline regression sample was re-divided into three regional sub-samples, East, Central, and West, to further analyze the impact of regional heterogeneity on the poverty reduction effect of public health services. (The criteria for dividing East, Central, and West were based on the level of economic development, natural resource status, and degree of policy preference according to the 2003 criteria of the National Bureau of Statistics of China. Among them, eastern provinces include Beijing, Tianjin, Hebei, Liaoning, Jiangsu, Shanghai, Zhejiang, Guangdong, Shandong, Hainan, and Fujian; central provinces include Anhui, Henan, Heilongjiang, Hubei, Hunan, Jilin, Jiangxi, and Shanxi; and western provinces include Guangxi, Guizhou, Inner Mongolia, Ningxia, Qinghai, Shaanxi, Sichuan, Yunnan, and Gansu.) The regression results of the subsamples are shown in Table 9. The results show that there is obvious regional heterogeneity in the impact of public health education and health file registration on the multidimensional poverty of migrant workers, and this poverty reduction effect has the characteristics of western> eastern > central. We try to give some possible explanations. The level of economic development and marketization in the eastern and central regions is relatively high [40], the employment security and welfare systems for migrant workers are more complete, and the migrant worker groups are less dependent on public health services. However, rural migrant workers in western rural areas generally have low incomes, and the construction of basic medical service facilities is relatively lagging. Then, the rigid demand of migrant workers for public health services will be significantly higher than that of the eastern and central regions, and public health services are needed to help them improve their health and alleviate poverty [41].

In summary, the impact of public health services on the multidimensional poverty of migrant workers is heterogeneous, and the improvement of public health services has the greatest effect on the multidimensional poverty alleviation of the western region, the new generation, and female migrant workers. Regarding the transmission mechanism of this poverty reduction effect, the hypothesis proposed in this paper based on the previous theoretical analysis is as follows: public health services can reduce the multidimensional poverty level of migrant workers by improving their health, reducing medical expenditures, increasing labor income, and enhancing their identity. Some evidence could provide support for these conjectures. Spadaro et al. (2013) found that Spanish public health services can influence the distribution of income and alleviate poverty [42]. Arthur and Oaikhenan (2017) found that the decline in mortality in sub-Saharan Africa was significantly influenced by public health spending [43]. However, due to the limitations of cross-sectional data samples and indicators, it is difficult to conduct empirical research on the abovementioned transmission mechanism in this paper, which is also the direction of our further research.

Nevertheless, we have some other findings. Focusing on other control variables (Table 3), we find that the gender of migrant workers is also significantly negatively correlated with their multidimensional poverty level, which shows that migrant female workers are more likely to fall into multidimensional poverty than male migrant workers. This is consistent with the research of Wang Y (2018). The reason for this difference may be their differences in human capital and gender discrimination [44]. From the perspective of age, as age increases, the possibility of migrant workers falling into multidimensional poverty increases. However, the age square coefficient is negative, indicating that the age of migrant workers has an “inverted U-shaped” relationship with their multidimensional poverty levels. In addition, control variables such as marriage, education, ethnicity, and occupational stability are all in line with expectations. It is worth noting that at the level of regional characteristics, the inflow of provincial capital cities will increase the possibility of migrant workers falling into multidimensional poverty [45]. This may be because although the nominal wages in large cities are higher, the impact of city size on the cost of living exceeds the benefits brought by nominal wages, especially the high costs of housing, medical care, and children’s education [46].

## 6. Conclusions

Based on CMDS data, this paper systematically investigated the role of public health services as the basic content of basic public services in alleviating the multidimensional poverty of migrant workers. Our analysis, in general, found that the current average value of the multidimensional poverty deprivation value of migrant workers is 0.1806. In addition, the public health service gaps remain relatively large. The proportions of migrant workers who have not established residents’ health files and who have not received public health education are 74.22% and 29.92%, respectively. Second, public health services can significantly alleviate the multidimensional poverty of migrant workers. After the IV-2SLS method is used to alleviate the potential endogeneity problems and the PSM method and other robustness tests, the conclusion is still valid. Third, the multidimensional poverty reduction of migrant workers by public health services is heterogeneous. In contrast, the improvement of public health services has the greatest effect on the multidimensional poverty alleviation of the western region, the new generation, and female migrant workers.

Overall, the research conclusions have certain policy implications. Migrant workers are a marginal group in the urban public services. When dealing with the multidimensional poverty problem of migrant workers in cities, we can think about promoting public health education for migrant workers and promoting health file registration, continuing to improve the construction of the public service system, and creating a favorable policy environment for the protection of migrant workers’ health and other interests. Specifically, first, it is necessary to promote the development of the equalization of public health services in stages and types according to the individual, time, and local conditions [47]. We need to respect, care for, and maximize the interests of migrant workers who are the least beneficiaries and reasonably weigh the tension between equal rights and special protection [48]. Second, we must accurately identify the group of poor migrant workers and dynamically monitor the multidimensional poverty level of migrant workers. In view of the different dimensions of poverty, the focus of public health service policy guidance should also be different [49]. Third, we need to refer to public health service policies and attach importance to public service policy support for migrant workers in the education and settlement of their children [50]. Compared with the social relief poverty alleviation for specific groups, the supply of basic public services and social services for a wider range of groups is more equitable. While improving the poverty of migrant workers, it is also conducive to reducing the welfare dependence of the adult working population [51].

## Figures and Tables

**Table 1:** Dimensions, indicators, and weights of multidimensional poverty measurement of migrant workers.

Dimension	Indicator	Explanation	Weight
Economy	Eco1: Difficulties with low income in current residence	Yes = 1; No = 0;	1/15
	Eco2: Difficulties in finding a stable job in the current residence	Yes = 1; No = 0;	1/15
	Eco3: Difficulties in doing business in the current residence	Yes = 1; No = 0;	1/15
Healthy	Hel: Your self-assessment of your physical health in the current residence	Healthy =0; Others =1;	1/5
Social Integration	Int1: Do you have any difficulties in your current residence that you are not used to?	Yes = 1; No = 0;	1/10
	Int2: Do you think the locals are unwilling to accept you?	Yes = 1; No = 0;	1/10
Life Expectancy	Exp1: Difficulties in not being able to buy a house in the current residence	Yes = 1; No = 0;	1/10
	Exp 2: Difficulties with children enrolling in the current residence	Yes = 1; No = 0;	1/10
Caring about Family	Care1: Difficulties in supporting the elderly in the hometown	Yes = 1; No = 0;	1/15
	Care2: Difficulties in caring for children in the hometown	Yes = 1; No = 0;	1/15
	Care3: Difficulties in spouse loneliness in the hometown	Yes = 1; No = 0;	1/15

**Table 2:** Summary statistics.

Variable Name	Definition	Mean	SE	Min	Max
Explained variable (dimension)					
PDL	Poverty deprivation levels of migrant workers	0.1806	0.1833	0	0.9333
Economy	Poverty level of migrant workers’ economic dimension	0.2685	0.3430	0	1
Healthy	Poverty level of migrant workers’ health dimension	0.1564	0.3632	0	1
Social integration	Poverty level of migrant workers’ social integration dimension	0.0687	0.1832	0	1
Life expectancy	Poverty level of migrant workers’ future development dimension	0.2711	0.3501	0	1
Family concern	Poverty level of migrant workers’ family concern dimension	0.1381	0.2196	0	1
Core explanatory variables					
Public health education	Number who received public health education	3.5358	3.3707	0	9
Health file registration	Not established = 0, established = 1	0.2578	0.4375	0	1
Human capital characteristics					
Gender	Female = 0, male = 1	0.5940	0.4911	0	1
Age	Age of respondent in 2017	35.7808	9.8497	15	79
Age2	Age × Age/100	13.7728	7.6126	2.25	62.41
Marriage	Unmarried = 0; married = 1	0.7964	0.4027	0	1
Education	Have not attended school = 0; elementary or junior high school = 1; high school = 2; university and above = 3	1.4800	0.7598	0	3
Party	CCP member = 1; others = 0	0.0384	0.1921	0	1
Ethnicity	Han nationality = 1; others = 0	0.9188	0.2731	0	1
Stability	Stable = 1; unstable = 0;	0.1727	0.3780	0	1
Family characteristics					
Family size	Family population	3.0326	1.2166	1	10
Social capital	Participation in activities such as trade unions, associations, classmate get-togethers, and fellow villagers’ associations	0.7469	1.0196	0	6
Regional characteristics					
lnGDP	Logarithmic value of provincial GDP in 2017	10.3574	0.8058	7.3450	11.5124
Capital city	Provincial capital city = 1; nonprovincial capital city = 0	0.4725	0.4992	0	1
Urban	Town = 1; countryside = 2	1.3409	0.4740	1	2

**Table 3:** Estimation results of the impact of public health services on the multidimensional poverty of migrant workers.

Variables	Explained Variable: PDL			
	(1)	(2)	(3)	(4)
Public health education	−0.0013 ***		−0.0014 ***	
	(0.0002)		(0.0002)	
Health file registration		−0.0150 ***		−0.0162 ***
		(0.0017)		(0.0017)
Gender			−0.0078 ***	−0.0079 ***
			(0.0016)	(0.0016)
Age			0.0076 ***	0.0076 ***
			(0.0006)	(0.0006)
Age2			−0.0071 ***	−0.0070 ***
			(0.0007)	(0.0007)
Marriage			0.0095 ***	0.0100 ***
			(0.0025)	(0.0025)
Education			−0.0136 ***	−0.0136 ***
			(0.0011)	(0.0011)
Party			−0.0016	−0.0014
			(0.0039)	(0.0039)
Ethnicity			−0.0329 ***	−0.0318 ***
			(0.0029)	(0.0029)
Family size			0.0190 ***	0.0189 ***
			(0.0008)	(0.0008)
Social capital			0.0008	0.0007
			(0.0008)	(0.0008)
Stability			−0.0035 *	−0.0037 *
			(0.0021)	(0.0021)
LnGDP			−0.0056 ***	−0.0053 ***
			(0.0010)	(0.0010)
Capital city			0.0053 ***	0.0049 ***
			(0.0016)	(0.0016)
Urban			−0.0173 ***	−0.0172 ***
			(0.0017)	(0.0017)
Cons	0.2333 ***	0.2328 ***	0.1106 ***	0.1072 ***
	(0.0036)	(0.0036)	(0.0153)	(0.0153)
Industry FE	YES	YES	YES	YES
Outflow areas FE	YES	YES	YES	YES
N	53,857	53,857	53,857	53,857
R2	0.0303	0.0310	0.0906	0.0914

Note: Standard errors of estimated coefficients are in parentheses. Significance relationships are shown as indicated by the *p*-values: * *p* < 0.10, *** *p* < 0.01.

**Table 4:** Estimated results based on IV-2SLS.

Variables	First Stage	Second Stage	First Stage	Second Stage
	(1) Public Health Education	(2) PDL	(3) Health File Registration	(4) PDL
IV1: Community public health attention	0.0734 ***	——	0.0082 ***	——
IV2: Policy cognition of migrant workers	2.3217 ***	——	0.3457 ***	——
Public health education	——	−0.0084 *** (0.0007)	——	——
Health file registration	——	——	——	−0.0566 *** (0.0046)
Human capital characteristics	YES	YES	YES	YES
Family characteristics	YES	YES	YES	YES
Regional characteristics	YES	YES	YES	YES
Industry FE	YES	YES	YES	YES
Outflow areas FE	YES	YES	YES	YES
First stage F value	722.31 ***	——	601.84 ***	——
Over-identification test *p*-value	——	0.5901	——	0.7667
N	53,153	53,153	53,153	53,153
R^2^	0.2136	0.0760	0.1874	0.0829

Note: Standard errors of estimated coefficients are in parentheses. Significance relationships are shown as indicated by the *p*-values: *** *p* < 0.01.

**Table 5:** Robustness test: average treatment effect (ATT).

Variable Name		ATT	Standard Error	*t* Test Value
Public health education	At least 1 public health education	−0.0042 *	0.0025	−1.6900
	At least 5 public health education	−0.0190 ***	0.0022	−8.7400
	Occupational disease prevention education	−0.0321 ***	0.0021	−15.0400
	Emergency public service education	−0.0149 ***	0.0021	−6.9800
Health file registration		−0.0212 ***	0.0022	−9.4600

Note: Standard errors of estimated coefficients are in parentheses. Significance relationships are shown as indicated by the *p*-values: * *p* < 0.10, *** *p* < 0.01.

**Table 6:** Robustness test: using binary explanatory variables.

Explained Variable	Poverty Status		Difficult Status	
Public health education	−0.0482 ***		−0.0474 ***	
	(0.0060)		(0.0049)	
Health file registration		−0.3230 *** (0.0405)		−0.3195 *** (0.0333)
Human capital characteristics	YES	YES	YES	YES
Family characteristics	YES	YES	YES	YES
Regional characteristics	YES	YES	YES	YES
Industry FE	YES	YES	YES	YES
Outflow areas FE	YES	YES	YES	YES
N	53,153	53,153	53,153	53,153

Note: Standard errors of estimated coefficients are in parentheses. Significance relationships are shown as indicated by the *p*-values: *** *p* < 0.01.

**Table 7:** The gender heterogeneous effect of public health services in alleviating poverty.

Explained Variable PDL	(1)	(2)	(3)	(4)
	Male Sample		Female Sample	
Public health education	−0.0079 *** (0.0009)	——	−0.0090 *** (0.0011)	——
Health file registration	——	−0.0526 *** (0.0060)	——	−0.0607 *** (0.0072)
Human capital characteristics	Yes	Yes	Yes	Yes
Family characteristics	Yes	Yes	Yes	Yes
Regional characteristics	Yes	Yes	Yes	Yes
Industry FE	Yes	Yes	Yes	Yes
Outflow areas FE	Yes	Yes	Yes	Yes
N	31,573	31,573	21,580	21,580
R^2^	0.0719	0.0795	0.0845	0.0904

Note: Standard errors of estimated coefficients are in parentheses. Significance relationships are shown as indicated by the *p*-values: *** *p* < 0.01.

**Table 8:** The intergenerational heterogeneous effect of public health services in alleviating poverty.

Explained Variable PDL	(1)	(2)	(3)	(4)
	New Generation Samples		Old Generation Sample	
Public health education	−0.0084 *** (0.0009)	——	−0.0082 *** (0.0011)	——
Health file registration	——	−0.0571 *** (0.0058)	——	−0.0543 *** (0.0074)
Human capital characteristics	Yes	Yes	Yes	Yes
Family characteristics	Yes	Yes	Yes	Yes
Regional characteristics	Yes	Yes	Yes	Yes
Industry FE	Yes	Yes	Yes	Yes
Outflow areas FE	Yes	Yes	Yes	Yes
N	31,954	31,954	21,199	21,199
R^2^	0.0655	0.0723	0.0314	0.0389

Note: Standard errors of estimated coefficients are in parentheses. Significance relationships are shown as indicated by the *p*-values: *** *p* < 0.01.

**Table 9:** The regional heterogeneous effect of public health services in alleviating poverty.

Explained Variable: PDL	(1)	(2)	(3)	(4)	(5)	(6)
	Eastern Sample		Central Sample		Western Sample	
Public health education	−0.0086 *** (0.0010)	——	−0.0047 *** (0.0014)	——	−0.0140 *** (0.0015)	——
Health file registration	——	−0.0595 *** (0.0067)	——	−0.0291 *** (0.0086)	——	−0.0927 *** (0.0096)
Human capital characteristics	Yes	Yes	Yes	Yes	Yes	Yes
Family characteristics	Yes	Yes	Yes	Yes	Yes	Yes
Regional characteristics	Yes	Yes	Yes	Yes	Yes	Yes
Industry FE	Yes	Yes	Yes	Yes	Yes	Yes
Outflow areas FE	Yes	Yes	Yes	Yes	Yes	Yes
N	26,596	26,596	12,214	12,214	14,343	14,343
R^2^	0.0672	0.0736	0.0848	0.0880	0.0919	0.0975

Note: Standard errors of estimated coefficients are in parentheses. Significance relationships are shown as indicated by the *p*-values: *** *p* < 0.01.

## Data Availability

Publicly available datasets were analyzed in this study. These data can be found here: http://www.ldrk.org.cn/ldrkdhcmrsf/front/index.html (accessed on 15 January 2020).
